# The two-component system CpxA/CpxR is critical for full virulence in *Actinobacillus pleuropneumoniae*

**DOI:** 10.3389/fmicb.2022.1029426

**Published:** 2022-10-05

**Authors:** Feng Liu, Qing Yao, Jing Huang, Jiajia Wan, Tingting Xie, Xuejun Gao, Diangang Sun, Fuxian Zhang, Weicheng Bei, Liancheng Lei

**Affiliations:** ^1^College of Animal Sciences, Yangtze University, Jingzhou, Hubei, China; ^2^School of Foreign Languages, Zhejiang Gongshang University, Hangzhou, Zhejiang, China; ^3^State Key Laboratory of Agricultural Microbiology, College of Veterinary Medicine, Huazhong Agricultural University, Wuhan, Hubei, China; ^4^College of Veterinary Medicine, Jilin University, Changchun, China

**Keywords:** *Actinobacillus pleuropneumoniae*, two-component system, CpxAR, polysaccharide capsule export, *cpxDCBA*

## Abstract

*Actinobacillus pleuropneumoniae*, a major bacterial porcine respiratory tract pathogen causing pig pleuropneumonia, has resulted in high economic losses worldwide. The mutation of the two-component system CpxAR strongly impacted the virulence of *A. pleuropneumoniae*, but the underlying regulatory mechanism remained unclear. Here, we found that CpxAR positively regulated the *cpxDCBA* gene cluster involved in polysaccharide capsule export. A capsular layer was confirmed in wild-type cells by transmission electron microscopy, whereas *cpxAR* and *cpxD* mutants were non-capsulated. The mutants for polysaccharide capsule export gene *cpxD* exhibited non-capsulated and were strongly impaired in virulence for mice, indicating a major role of CPS export system in virulence. We then demonstrated that CpxR directly regulated the transcription of the CPS export gene cluster *cpxDCBA*. Taken together, our data suggested that CpxAR is a key modulator of capsule export that facilitates *A. pleuropneumoniae* survival in the host.

## Introduction

*Actinobacillus pleuropneumoniae* is a Gram-negative facultative anaerobic bacterium belonging to the *Pasteurellaceae* family (Sassu et al., 2018). This pathogen is the aetiological agent of porcine pleuropneumonia in pigs of all ages, which is a highly contagious and often deadly respiratory disease causing substantial economic losses in the swine industry worldwide (Cohen et al., 2021). *A. pleuropneumoniae* colonizes the tonsils and nasal cavities of infected pigs, and transmission from pig to pig occurs mainly by respiratory droplets or direct contact (Bosse et al., 2002). It is currently classified into 19 serovars based on the antigenic properties of their capsular polysaccharides and lipopolysaccharides (Stringer et al., 2021). Previous studies have reported that many virulence factors play an important role in the pathogenicity of *A. pleuropneumoniae*, such as lipopolysaccharide, Apx toxins, polysaccharide capsule, and iron acquisition proteins (Loera-Muro et al., 2021).

Capsule polysaccharide (CPS) is encoded by the genes of the CPS biosynthetic locus *cpsABCD* and the CPS export locus *cpxDCBA* in *A. pleuropneumoniae* (Hathroubi et al., 2016). Unlike the former, the CPS export genes, *cpxDCBA*, are conserved in different *A. pleuropneumoniae* serovars (Bandara et al., 2003). The four genes encode an outer membrane lipoprotein (CpxD), a cytoplasmic membrane protein (CpxC), an integral membrane protein (CpxB), and an ATP-binding protein (CpxA) (Ward and Inzana, 1997).

Two-component signaling systems (TCSs), usually comprising a sensor histidine kinase (HK) and a cytoplasmic response regulator (RR), are involved in bacterial colonization and virulence through sensing environmental stimulus and responding accordingly (West and Stock, 2001; Li et al., 2018). Once it senses a physical or chemical signal, the histidine kinase will lead to autophosphorylation. The phosphoryl group is then transferred to the response regulator, which subsequently binds the target DNA promoters to alter gene expression (Gu et al., 2020). The genome of *A. pleuropneumoniae* encodes 5 paired of two-component systems, such as CpxAR, QseBC, ArcAB, PhoBR, and NarPQ (Xu et al., 2008).

The well-known TCS CpxAR, including the sensor histidine kinase CpxA, the cytopalsmic response regulator CpxR and the accessory protein CpxP, is required for sensing and coordinating the response to the envelope stress in *E. coli* (Raivio and Silhavy, 1997; Xu et al., 2014). In *A. pleuropneumoniae*, the Cpx system is composed of the histidine kinase CpxA and response regulator CpxR, and their genes constitute the *cpxRA* gene cluster (Li et al., 2018). Previous studies showed that the CpxAR system is a pleiotropic TCS in *A. pleuropneumoniae*, and involved in biofilm formation, oxidative stress, osmotic stress, heat stress, and virulence (Li et al., 2018; Yan et al., 2020). Li et al. found that CpxAR affects biofilm formation by regulating the expression of the *pgaABCD* operon through *rpoE* in *A. pleuropneumoniae* (Li et al., 2018). CpxAR has been reported to contribute to the virulence of *A. pleuropneumoniae* by altering O-antigen repeating unit biosynthesis (Yan et al., 2020). However, more underlying mechanisms of the CpxAR-mediated pathogensis of *A. pleuropneumoniae* remain to be elucidated. Here, the principal objective of this study is to reveal that the regulatory mechanism employed by CpxAR contributes to *A. pleuropneumoniae* virulence.

## Materials and methods

### Bacterial strains, culture conditions, plasmids, and primers

For this study, we used *A. pleuropneumoniae* strain S4074 as a representative strain. *A. pleuropneumoniae* strain was routinely grown at 37°C with shaking (180 rpm) in tryptic soy broth (TSB) medium (Solarbio, Beijing, China) supplemented with 10 μg/ml Nicotinamide Adenine Dinucleotide (NAD; Biofroxx, Einhausen, Germany) and 10% (vol/vol) newborn bovine serum (FBS; EVERY GREEN, Hangzhou, China). *Escherichia coli* strain BL21 was grown at 37°C with shaking (180 rpm) in Luria-Bertani (LB) medium. *E. coli* β2155 was grown in LB medium supplemented with 50 μg/ml diaminopimelic acid (Sigma-Aldrich, St. Louis, United States). We added 5 μg/ml chloramphenicol, or 50 μg/ml kanamycin as required. All strains and plasmids used in this study are listed in Table 1, and primers (Sangon Biotech Co., Ltd., Shanghai, China) are shown in Table 2.

**Table 1:** Bacterial strains and plasmids used in this study.

Strains/plasmids	Characteristics	Source/reference
*A. pleuropneumoniae*		
S4074	*A. pleuropneumoniae* reference strain of serovar 1; WT strain	From Prof. Weicheng Bei
*ΔcpxAR*	*A. pleuropneumoniae* 4,074 *cpxAR*-deletion mutant	From Prof. Weicheng Bei
*ΔcpxD*	*A. pleuropneumoniae* 4,074 *cpxD*-deletion mutant	This study
*CΔcpxAR*	Complemented strain of *ΔcpxAR*; Cm^r^	From Prof. Weicheng Bei
*CΔcpxD*	Complemented strain of *ΔcpxD*; Cm^r^	This study
*E. coli*		
*DH5a*	Cloning host for recombinant vector	Takara
*β2155*	Transconjugation donor for constructing mutant strain	From Prof. Weicheng Bei
*Plasmid*		
pEMOC2	Transconjugation vector: ColE1 ori mob RP4 sacB, Amp^r^Cm^r^	From Prof. Weicheng Bei
pEΔ*cpxD*	Up- and down-stream arms of *cpxD* were ligated sequentially into pEMOC2, and used as the transconjugation vector for *cpxD* gene deletion	This study
pJFF224-XN	*E. coli*-APP shuttle vector: RSF1010 replicon; mob oriV, Cm^r^	From Prof. Weicheng Bei
pCΔ*cpxD*	pJFF224-XN carrying the intact *cpxD*	This study
pET-30a	Expression vector; Kan^r^	Novagen
pET30a-*cpxR*	pET-30a carrying cpxR gene	This study

Cm^r^, Chloramphenicol resistance; Amp^r^, Ampicillin resistance; Kan^r^, Kanamycin resistance.

**Table 2:** Primers used in this study.

Primer	Sequence (5′–3′) a	Use
*cpxD*-S-F	CTGTCGACTCTTGTCTCGTCTCATCCAGCCACT	Amplification of *cpxD* upstream homology arms
*cpxD*-S-R	ATTCAGACAACGGCGCATTTATCCGAACTTTGTGAATTAGCCTCT	
*cpxD*-X-F	AGAGGCTAATTCACAAAGTTCGGATAAATGCGCCGTTGTCTGAAT	Amplification of *cpxD* downstream homology arms
*cpxD*-X-R	ATGCGGCCGCCGGTTTGCTTGGCTGACTGA	
*cpxD*-W-F	GCTAGTTTGGCTGCCTGCTC	Detection exterior of *cpxD* mutants
*cpxD*-W-R	TGTTCCGCAAATGAAATGGT	
*cpxD*-N-F	TTACCGTGCCGTTCGTGG	Detection interior of *cpxD* mutants
*cpxD*-N-R	GCAGCAACCGCATCTAATACAC	
*cpxD*-C-F	CCGCTCGAGTAATGCTTATCTGTTGAACCCTCCT	Amplification of *cpxD*
*cpxD*-C-R	AAGGAAAAAAGCGGCCGCTTAATAGGCACGAACGGCATTGGTC	
*cpxD*-F	GTATTCCGTCACGTGCCTTT	Detection the transcription of *cpxD*
*cpxD*-R	GCATTGGGAAACGCTGTAAT	
*cpxC*-F	ATTTCATTTGCGGAACAAGC	Detection the transcription of *cpxC*
*cpxC*-R	CGCATAAGCAATGCATCAAC	
*cpxB*-F	GCTACCAAGCCAAGCTCAAC	Detection the transcription of *cpxB*
*cpxB*-R	TTGCGGTTCGATTCCTTTAC	
*cpxA*-F	GGCAGTTTAACCGGTATGGA	Detection the transcription of *cpxA*
*cpxA*-R	CGAGAGTCACCTACCGCAAT	
16SrRNA-F	CCATGCCGCGTGAATGA	Detection the transcription of 16SrRNA
16SrRNA-R	TTCCTCGCTACCGAAAGAACTT	
*cpxD*-EMSA-F	TAATGCTTATCTGTTGAACCCTCCT	Amplification of *cpxD* promoter region for EMSA
*cpxD*-EMSA-R	CCCCAAAGAAAGGAGTAATCTAAGT	
*rpoE*-EMSA-F	TAAAAAGATAAGATAAGCGGTC	Amplification of *rpoE* promoter region for EMSA
*rpoE*-EMSA-R	AGTGTGTAACAAAAATGAAAAGT	
*rpoD*-EMSA-F	GCGGAAGAAAAGCAAGAGTTGGTCA	Amplification of *rpoD* promoter region for EMSA
*rpoD*-EMSA-R	TCCATAATTGTATCCGTTTTGTGTG	

The pEMOC2 suicide plasmid was used to construct the mutant strain Δ*cpxD* following an allelic exchange methodology, as described earlier (Liu et al., 2018). The complementation strain CΔ*cpxD* was generated using the shuttle plasmid pJFF224-XN as previously described (Liu et al., 2018). The Δ*cpxD* mutant and complementation strain CΔ*cpxD* were verified by PCR (Supplementary Figure 1) and sequencing.

### RNA extraction, qRT-PCR, and RT-PCR

RNA extraction and qRT-PCR assays were performed as described earlier, with some modifications (Huang et al., 2018). The wild-type and ∆*cpxAR* mutant strains were grown in 5 ml of TSB overnight, normalized to an optical density of 600 nm (OD600) of 0.05, and incubated at 37°C with shaking (180 rpm) to an OD600 of 0.6. After the cells have grown to an OD600 of 0.6, cells were centrifuged at 4°C for 5 min at 10, 000 *g*, treated with the Bacteria Total RNA Isolation Kit (Sangon Biotech, Shanghai, China), and stored at −80°C until analysis. Reverse transcription was performed using the HiScript II first-strand cDNA synthesis kit (Vazyme, Nanjing, China). qRT-PCR was performed using the ViiA-7 Real-Time PCR System (Applied Biosystems, Waltham, United States) and SYBR qPCR Mix (Vazyme, Nanjing, China). Fold change data were normalized according to the housekeeping gene 16S rRNA, and analyzed using the 2^−ΔΔCt^ method (Livak and Schmittgen, 2001). RT-PCR acrossing the *cps2A-cpxD*, *cpxD-cpxC*, *cpxC-cpxB*, and *cpxB-cpxA* junctions was conducted as described previously (Xiong et al., 2019; Cheng et al., 2021). Amplified RT-*PCR products were* electrophoresed and photographed using a Gel Image Analyzing JS-1800 system (Peiqing, China).

### Protein expression and purification

The PCR-amplified *cpxR* gene from *A. pleuropneumoniae* strain S4074 was cloned into the pET30a vector by digesting with Nde I and Xho I. Then, the CpxR expression plasmid was transformed into *E. coli* BL21(DE3), and their expression was induced by the addition of 0.5 mM IPTG and incubated at 16°C overnight. The cells were disrupted using a Ultrasonic Homogenizer (SCIENTZ, Ningbo, China), and centrifuged at 12,000 *g* for 20 min to remove cellular debris. The supernatant was extracted with Ni-nitrilotriacetic acid (Ni-NTA) resin affinity chromatography following the manufacturer’s instructions. The purified protein concentration was determined by a BCA protein assay kit (Beyotime, Shanghai, China), and the purity was checked by 12% SDS-PAGE.

### Electrophoretic mobility shift assay

To study the binding of CpxR to the DNA probes, the electrophoretic mobility shift assays (EMSAs) were performed using a Chemiluminescent EMSA Kit (Beyotime, Shanghai, China) as previously described (Cheng et al., 2021). The DNA probes were generated by PCR amplification from 1 to 196 bp upstream of the start codon of *cpxD* gene, purified using a Gel Extraction Kit (Omega, Norcross, United States), and labeled using a EMSA Probe Biotin Labeling Kit (Beyotime, Shanghai, China). The recombinant protein CpxR was phosphorylated *in vitro* by 50 mM acetyl phosphate (Sigma, St. Louis, United States) (Pogliano et al., 1997). Increasing amounts of phosphorylated CpxR protein (0 to 4 pmol) were incubated with the labeled probe (1 μM) in binding buffer (50 mM Tris (pH 8.0), 100 mM KCl, 2.5 mM MgCl2, 0.2 mM dithiothreitol (DTT), 2 μg salmon sperm DNA, 10% glycerol) for 20 min at room temperature. The reaction mixtures were directly subjected to 4% non-denaturing polyacrylamide electrophoresis. The gel was transferred to a nylon membrane (Beyotime, Shanghai, China), and imaging was performed using JS-1070 fluorescent chemiluminescence gel imaging system (Peiqing, Shanghai, China).

### DNase I footprinting assay

For preparation of fluorescent 6-carboxyfluorescein (FAM) labeled probe, the *cpxD* promoter was amplified from 1 to 196 bp upstream of the start codon by PCR using the plasmid pEASY-cpxD as a template and primers of M13F (FAM) and M13R. For each assay, the 6-FAM-labeled probe (400 ng) was mixed with different amounts of phosphorylated CpxR protein in a 40 μl reaction volume for 30 min at 25°C. Subsequently, the mixture was incubated with 0.015 unit DNase I (Promega, Madison, United States) at 37°C for 1 min. The reaction was terminated by adding 140 μl DNase I stop solution (200 mM unbuffered sodium acetate, 30 mM EDTA and 0.15% SDS). The samples were extracted by phenol/chloroform, and the pellets containing DNA were dissolved in 30 μl water. The results were analyzed using 3130xl DNA analyzer and Peak Scanner software v1.0 (Applied Biosystems, Waltham, United States).

### Transmission electron microscope

For transmission electron microscope (TEM), *A. pleuropneumoniae* strain was grown on TSA plates overnight at 37°C, harvested by centrifugation (5,000 *g*, 4°C, 5 min), and then incubated in FBS (EVERY GREEN, Hangzhou, China) for 30 min at 37°C. The samples were fixed for 2 h with 2.5% glutaraldehyde and placed onto 200 mesh copper grids. Subsequently, the copper grids were air-dried and observed with a 120KV biological transmission electron microscope (HITACHI, Tokyo, Japan).

### Animal test

In all experiments, Kunming (KM) mice (female, 6 weeks old) purchased from CTGU University Laboratory Animal Center (Quality Certificate No. 42010200007283) were used. All animal experiments were approved by the Animal Ethics Committee of the Yangtze University. To investigate the survival curves of the mice, 1 × 10^7^ CFUs of WT S4074, ∆*cpxAR*, ∆*cpxD*, C∆*cpxAR* or C∆*cpxD* was injected *via* the abdominal cavity of each mouse (8 mice per group). The survival rates were recorded daily in 1 week after challenge.

In addition, to evaluate the colonization ability, another 5 groups (8 mice/group) were given intraperitoneal injections with 1 × 10^7^ CFUs of WT S4074, ∆*cpxAR*, ∆*cpxD*, C∆*cpxAR* or C∆*cpxD*. At 8 h after injection, one half of the lung and liver from each mice was collected aseptically, weighed, homogenized, diluted serially, and plated to determine bacterial counts. The remaining lungs were fixed in tissuse’s fixative (Biosharp, Beijing, China) at 4°C for histo pathological analysis as described earlier (Guo et al., 2018).

### Bioinformatic and statistical analysis

The promoter and the transcriptional start site of the *cpxD* gene were, respectively, predicated by using BPROM^1^ and BDGP.^2^ Two-tailed Student’s *t* tests were used to analyze the significance between various mutants and WT strain using GraphPad Prism version 7.0 (GraphPad, La Jolla, United States). Values of *p* < 0.05 was considered statistically significant.

## Results

### CpxAR is required for capsule synthesis in *Actinobacillus pleuropneumoniae*

**Figure 1:**
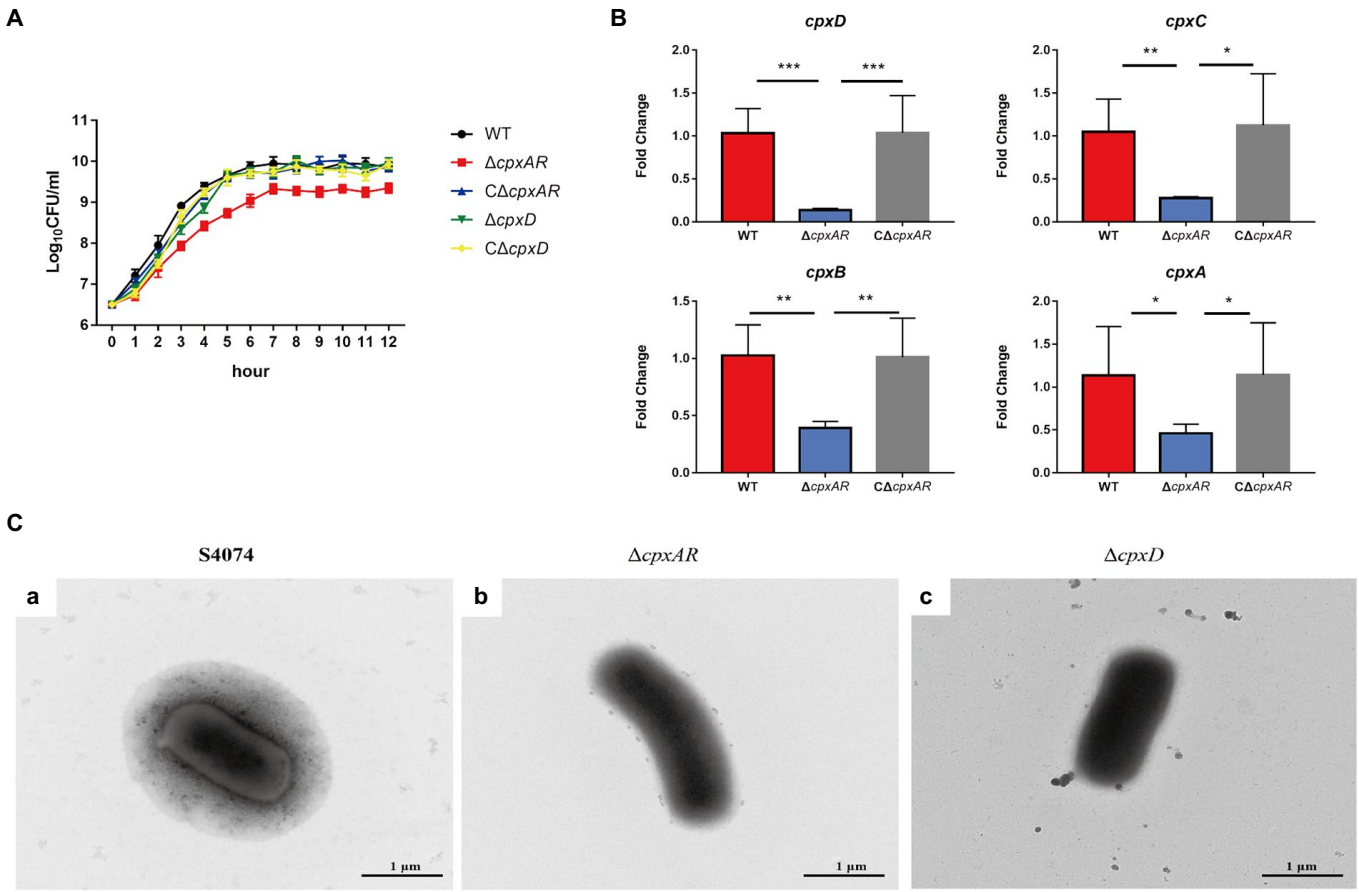
CpxAR impacts the capsule production. **(A)** The growth analysis of the WT S4074, ∆*cpxAR*, C∆*cpxAR*, ∆*cpxD*, and C∆*cpxD* strains. **(B)** The relative mRNA levels of *cpxD, cpxC, cpxB, and cpxA (capsular polysaccharide export gene) in* the Δ*cpxAR* mutant compared to the parent strain *determined by* RT-qPCR. *p < 0.05, **p < 0.01, ***p < 0.001. **(C)** Observation of the capsule layer of the WT S4074 **(A)**, ∆*cpxAR* **(B)**, ∆*cpxD* **(C)**, strains by TEM.

The growth traits of the WT S4074, ∆*cpxAR*, C∆*cpxAR*, ∆*cpxD,* and C∆*cpxD* strains were investigated. As shown in Figure 1A, only the ∆*cpxAR* mutant exhibited growth defects, which was consistent with previous studies (Li et al., 2018). To elucidate the mechanism by which CpxAR impacts the pathogenicity of *A. pleuropneumoniae*, qRT-PCR was performed to identify the genes regulated by CpxAR. The qRT-PCR analysis showed that the relative transcript levels of the four CPS export genes, *cpxD*, *cpxC*, *cpxB,* and *cpxA* (capsular polysaccharide export gene), were significantly decreased in the ∆*cpxAR* mutant strain (Figure 1B). These data indicated that CpxAR regulate the expression of the four CPS export genes in *A. pleuropneumoniae.*

Previous work on *A. pleuropneumoniae* found that the WT S4074 strain forms a extensive layer of capsular material covering the cells (Jacques et al., 1988). Here, we observed an extensive layer of capsular material covering the WT S4074 as expected, whereas ∆*cpxAR* and ∆*cpxD* mutant strains were not found layer of capsular material (Figure 1C). These data indicated that CpxAR contributes to capsule synthesis in *A. pleuropneumoniae*.

### Characterization of the *cpxDCBA* operon in *Actinobacillus pleuropneumoniae*

**Figure 2:**
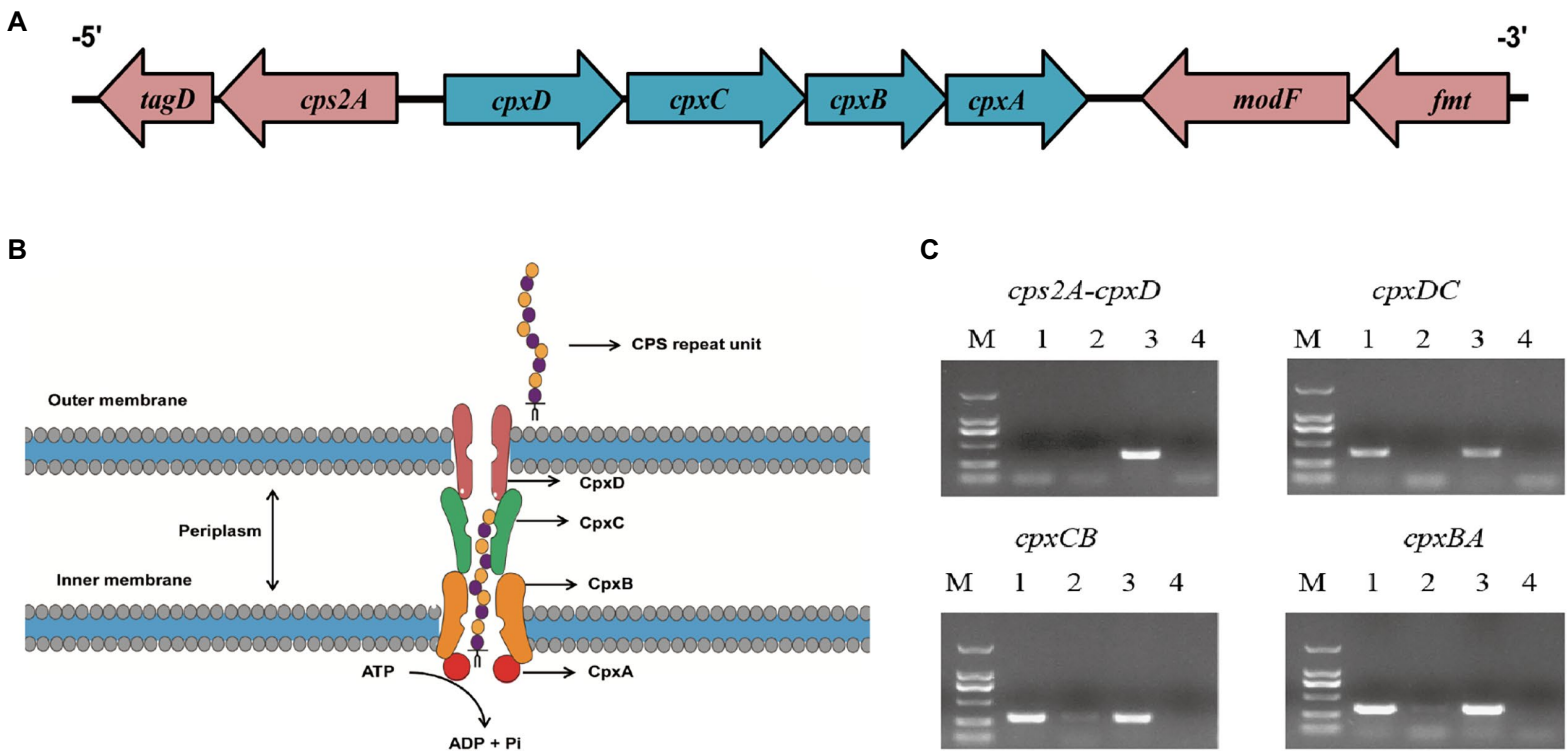
The *cpxD*, *cpxC*, *cpxB,* and *cpxA* genes form a single operon. **(A)** Schematic presentation of the capsule export locus. **(B)** Diagram showing the role of CpxD, CpxC, CpxB, and CpxA (capsular polysaccharide export protein) in CPS synthesis. **(C)** RT-PCR analysis confirmed that the *cpxD*, *cpxC*, *cpxB,* and *cpxA* (capsular polysaccharide export gene) genes form an operon. The Lane 1–4 were cDNA, total RNA, genomic DNA, and no-template control, respectively.

These four capsule export genes, including *cpxD*, *cpxC*, *cpxB,* and *cpxA* (capsular polysaccharide export gene), are in the chromosome, and such cluster is adjacent to the gene *cps2A* (Figures 2A,B). To verify whether these four genes are controlled by one promoter, we performed RT-PCR across the *cps2A-cpxD*, *cpxD-cpxC*, *cpxC-cpxB*, and *cpxB-cpxA* junctions. The RT-PCR analysis indicated that the *cpxDCBA* gene cluster is a single operon (Figure 2C).

### CpxR binds specifically to the *cpxD* promoter region

**Figure 3:**
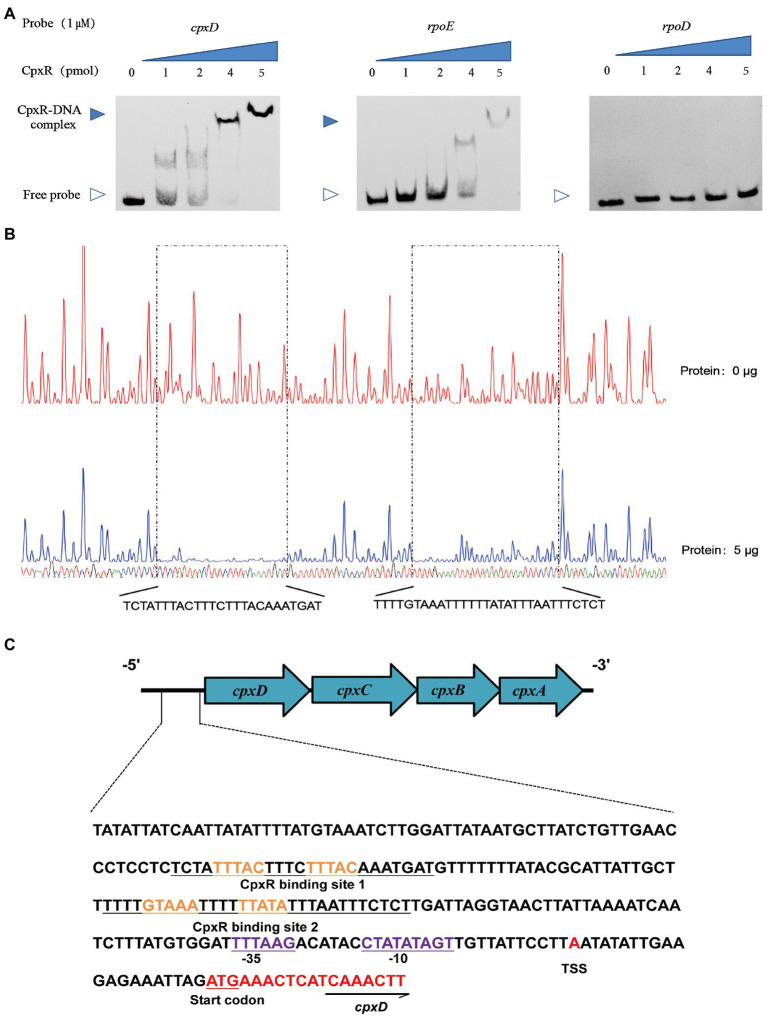
CpxR binds to the *cpxD* promoter region. **(A)** EMSAs performed with various concentrations of phosphorylated CpxR (0–4 pmol) and the promoter regions of *cpxD*, *rpoE* (positive control) and *rpoD* (negative control). **(B)** Mapping the CpxR binding sites in the *cpxD* promoter by DNase I footprinting. Protected regions were shown below the footprinting results. **(C)** Nucleotide sequences of *cpxD* promoter. CpxR-binding site was shown in blue nucleotides boxed in black, and − 35 box, −10 box were underlined and shown in green. Start codon of *cpxD* was underlined and shown in red, and the TSS was shown in red.

To investigate the mechanism of CpxAR-mediated transcriptional regulation of the *cpxDCBA* operon, we analyzed the binding site for CpxR in the upstream region of *cpxDCBA* operon using EMSA. EMSAs showed that the CpxR protein could bind to the promoter region of the *cpxDCBA* operon (Figure 3A). To further analyze the CpxR-*cpxD* interaction, DNase I footprinting was used to map the precise binding site. Two CpxR-binding sites were found 92 to 121 bp and 146 to 170 bp upstream of the start codon, and the sequences were 5’-TCTATTTACTTTCTTTACAAATGAT-3′ and 5’-TTTTGTAAATTTTTTATATTTAATTTCTCT-3′ respectively (Figure 3B). Collectively, these findings indicated that CpxR directly regulates the expression of *cpxDCBA* operon.

To obtain a more detailed picture of the CpxR binding site, the promoter and the transcription start site of the *cpxD* gene were, respectively, predicted by BPROM and BDGP. The *cpxD* transcriptional start site, designated as TSS, was detected 21-bp upstream of the start codon, and determined as A (Figure 3C). In addition, we performed a bioinformatic search in the promoter region of *cpxD* and identified a putative ˗10 CTATATAGT box and a putative ˗35 TTTAAG box, respectively, located 33 bp and 48 bp upstream of the start codon (Figure 3C).

### CpxAR and *cpxD* are involved in virulence for mice

**Figure 4:**
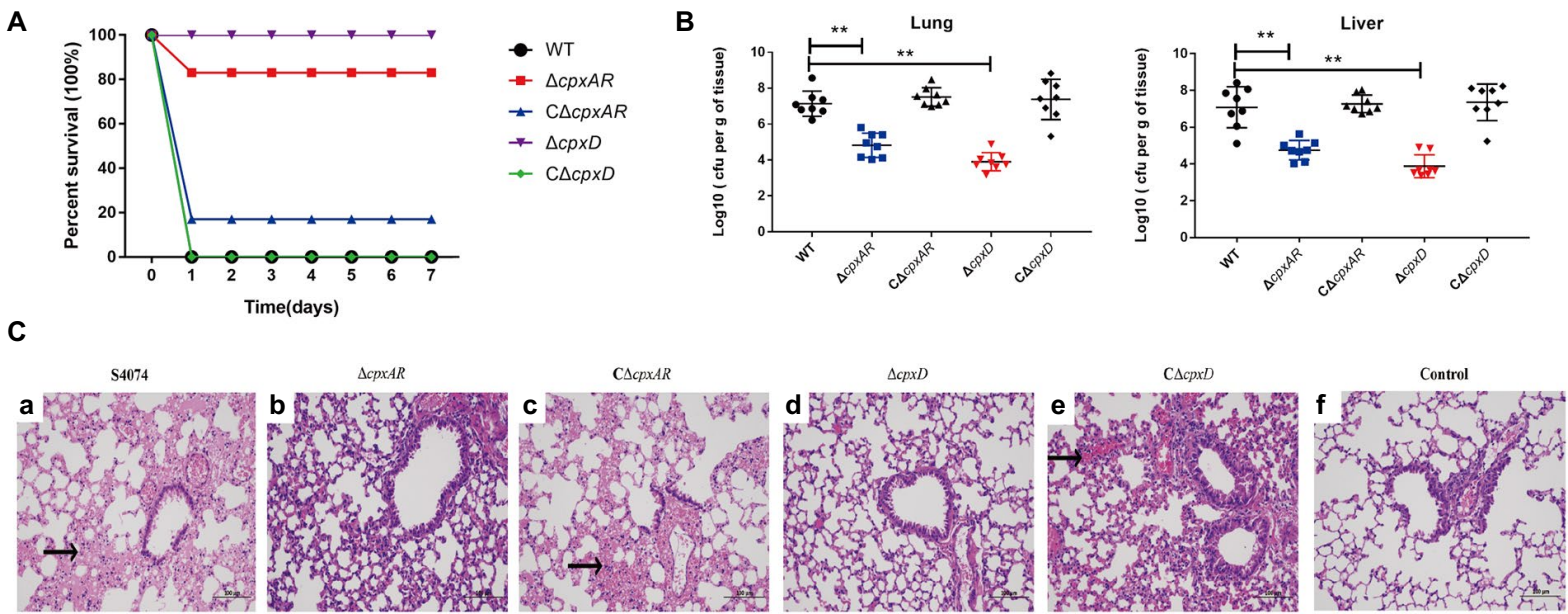
CpxAR and CpxD contribute to the virulence and colonization of *A. pleuropneumoniae*. The survival rates **(A)**, bacterial loads in the lungs and livers **(B)**, and histopathology of the lung tissues **(C)** of Kunming mice challenged with the WT S4074 (a), ΔcpxAR (b), *ΔcpxD* (c), C*ΔcpxAR* (d), and C*ΔcpxD* (e) strains at a dose of 1 × 10^7^ CFU/mouse. The control group (f) was only injected with 100 µl of PBS **p < 0.01.

To verify whether the CpxAR-*cpxDCBA* pathway plays an important role in the virulence of *A. pleuropneumoniae*, the WT S4074, ∆*cpxAR*, C∆*cpxAR*, ∆*cpxD,* and C∆*cpxD* strains were further compared through survival and colonization assays *in vivo* in KM mice. As shown in Figure 4A, the mice infected by the ∆*cpxAR* and ∆*cpxD* strain showed significantly higher survival rate of 83 and 100% respectively; whereas, the mice infected by the WT, C∆*cpxAR* and C∆*cpxD* strains showed the survival rate of 0, 17 and 0%, respectively. As shown in Figure 4B, the bacterial loads in the lungs and livers of ∆*cpxAR*- and ∆*cpxD*-infected mice were significantly lower than that of WT-, C∆*cpxAR*- and C∆*cpxD*-infected mice.

Histologic examination of the lung tissue sections from all of the mice infected with WT, C∆*cpxAR* and C∆*cpxD* strains showed the classic features of pneumonia, such as hyperemia, swelling, hemorrhage, edema, consolidation, but this was not evident in ∆*cpxAR*- and ∆*cpxD*-infected mice (Figure 4C). Taken together, these observations indicated that the CpxAR-*cpxDCBA* pathway contributs to the pathogenesis of *A. pleuropneumoniae*.

## Discussion

Two-component system is one of the key bacterial mechanisms that enables bacteria to sense and respond to host stimulation, which is critical for the pathogenic process (Matanza et al., 2021). The CpxAR system is the principal determinant of many biological processes in *A. pleuropneumoniae*, such as biofilm formation, heat stress and O-antigen repeating unit biosynthesis (Li et al., 2018; Yan et al., 2020). Previous studies have found that CpxAR is required for virulence in *A. pleuropneumoniae* (Li et al., 2018; Yan et al., 2020). However, the crucial role of CpxAR in the pathogenesis of *A. pleuropneumoniae* requires further investigation. In this study, we explored whether CpxAR-regulated genes are critical to the successful infection of *A. pleuropneumoniae*.

CPS is one of the major virulence factors of *A. pleuropneumoniae*, which can protect the bacteria from the host’s immune response, such as phagocytic uptake and complement-mediated bacteriolysis (Budde et al., 2020). The thickness of the capsule is related to the virulence of *A. pleuropneumoniae*, and generally, the thicker the capsule, the more virulent the strain (Dubreuil et al., 2000). However, the regulation of capsule production in *A. pleuropneumoniae* is still unknown. In this study, TEM confirmed that the capsule layer of the Δ*cpxAR* strain was difficult to observe. These observations identified that CpxAR is a capsule regulator, which has been implicated in controlling the gene expression and production of CPS in *A. pleuropneumoniae*.

Previous studies showed that the sequence GTAAA-(N)_4–8_-GTAAA, or TTTAC-(N)_4–8_-TTTAC is the binding consensus sequence of CpxR (Keilwagen et al., 2009; Srinivasan et al., 2012; Feldheim et al., 2016; Tian et al., 2016). Here, two putative CpxR-binding sequence (TTTAC-N_4_-TTTAC and GTAAA-N_4_-TTATA) were, respectively, located 39–68 bp and 94–118 bp upstream of the promoter −35 region of the *cpxD* gene. Furthermore, we found that CpxR could directly bind to the *cpxD* promoter region by EMSA, and identified two CpxR-binding sites by DNase I footprinting which were consistent with the two putative sequences. In general, the CpxR-binding site is located upstream of the promoter region and activates their transcription (Raffa and Raivio, 2002). Previous work identified only one CpxR-binding site, but we found two in this study. The reason and the function of the two sites is worthy of further investigation.

**Figure 5:**
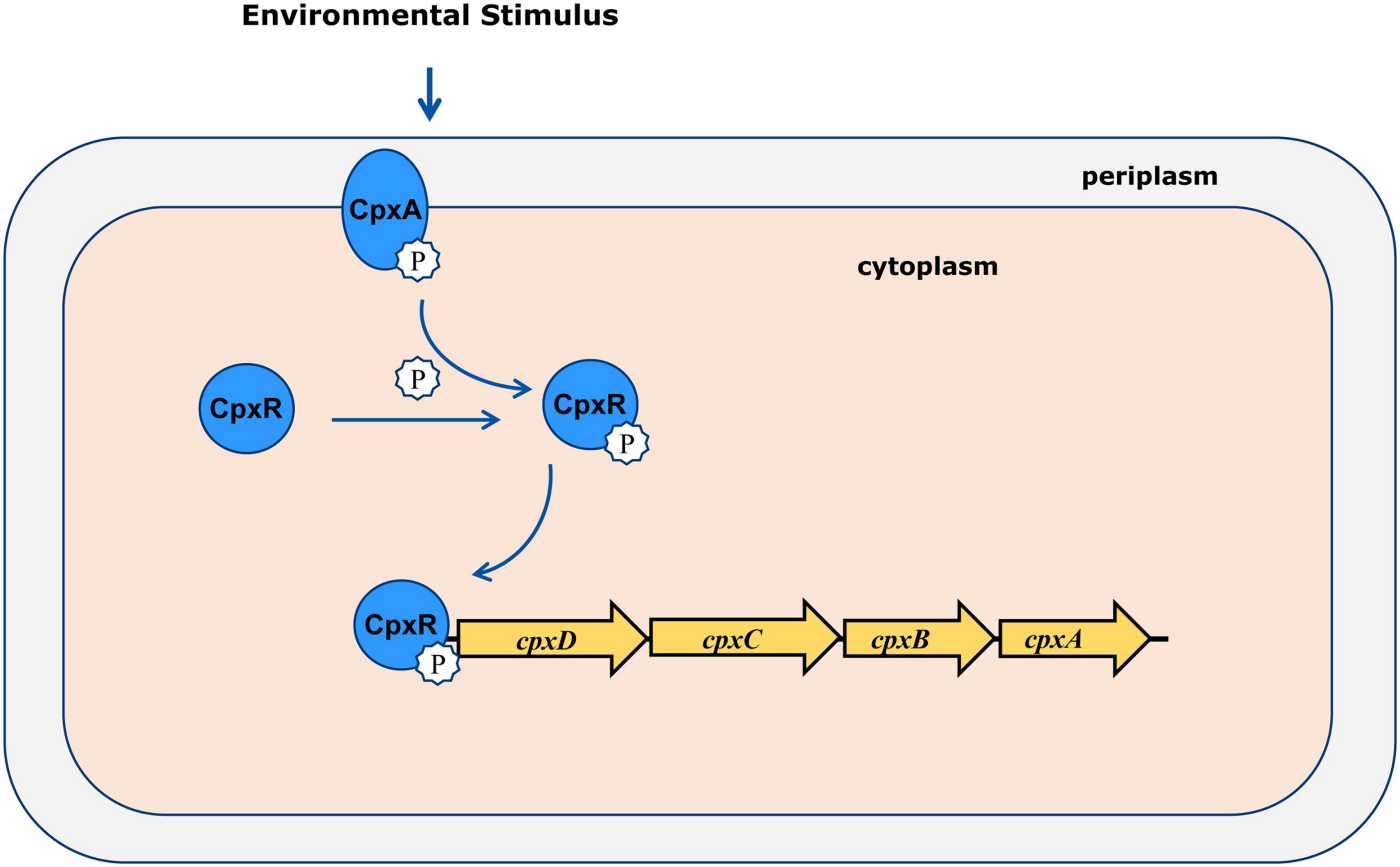
Molecular model of virulence regulation by CpxAR. *CpxDCBA* transcription is activated by CpxR-P, which contributes to the virulence in *A. pleuropneumoniae*.

In the present study, we showed that CpxAR positively regulates the CPS export operon *cpxDCBA* using qRT-PCR. Furthermore, EMSA and DNase I footprinting demonstrated that CpxR directly regulated the *cpx* operon by binding to the *cpxD* promoter region. In addition, these animal challenge tests showed that CpxD plays an important role in the virulence of *A. pleuropneumoniae*. Previous studies have shown that an insertion mutant in *cpxC*, encoding a cytoplasmic membrane protein which is essential for polysaccharide transport, caused less mortality in pigs compared to the parent strain (Rioux et al., 2000). These findings indicated that CpxAR contributes to the virulence of *A. pleuropneumoniae* by directly regulating the CPS export locus, *cpxDCBA* (Figure 5).

Our results showed that CpxAR plays a contributing role in virulence by affecting capsule synthesis through directly regulating the expression of the *cpxDCBA* operon. The CpxAR and CPS are present in many bacteria (Vogt and Raivio, 2012; Whitfield et al., 2020). Therefore, our findings may not only contribute to the understanding of the pathogenesis of *A. pleuropneumoniae*, but also to other bacteria. Future studies will focus on identifying more virulence factors regulated by CpxAR in *A. pleuropneumoniae*.

## Data availability statement

The original contributions presented in the study are included in the article/Supplementary material, further inquiries can be directed to the corresponding authors.

## Ethics statement

The animal study was reviewed and approved by the Animal Ethics Committee of the Yangtze University.

## Author contributions

FL, WB, and LL: conceived and designed the experiments. QY, JW, and TX: performed the experiments. FL and QY: analyzed the data. XG, DS, and FZ: contributed reagents, materials, and analysis tools. JH: polished the language. All authors contributed to the article and approved the submitted version.

## Conflict of interest

The authors declare that the research was conducted in the absence of any commercial or financial relationships that could be construed as a potential conflict of interest.

## Publisher’s note

All claims expressed in this article are solely those of the authors and do not necessarily represent those of their affiliated organizations, or those of the publisher, the editors and the reviewers. Any product that may be evaluated in this article, or claim that may be made by its manufacturer, is not guaranteed or endorsed by the publisher.

## Funding

This research was supported by the National Natural Science Foundation of China (32002252).

## Footnotes

^1^ http://linux1.softberry.com/berry.phtml?topic=bprom&group=programs&subgroup=gfindb

^2^ https://www.fruitfly.org/seq_tools/promoter.html
