# Supplementary figures and images for: The two-component system CpxA/CpxR is critical for full virulence in Actinobacillus pleuropneumoniae
Source: Front Microbiol. 2022 Oct 5;13:1029426. doi: 10.3389/fmicb.2022.1029426 (PMC9615922; doi:10.3389/fmicb.2022.1029426)

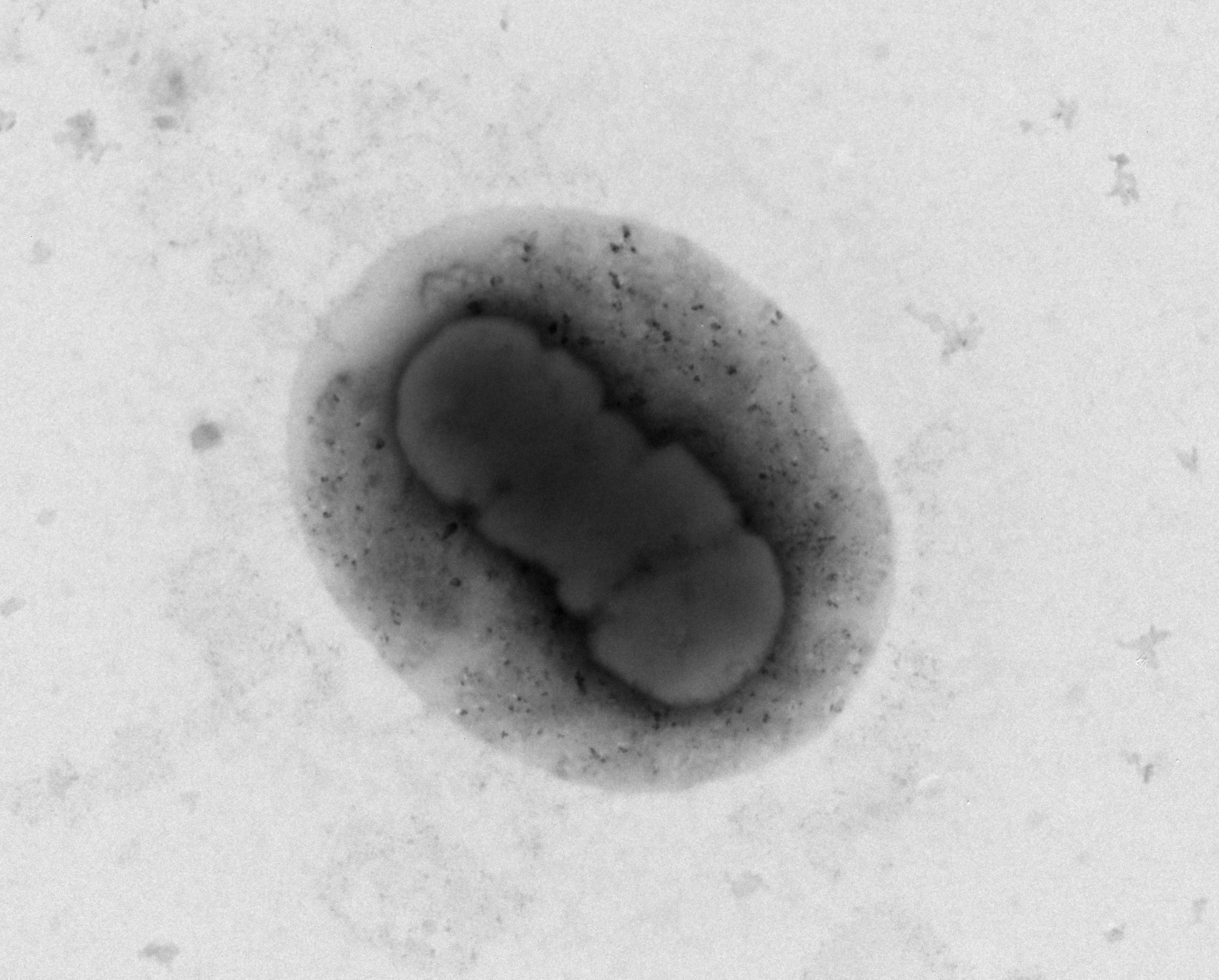

Supplement: Supplementary file 1 [file Data_Sheet_1.ZIP › original data/original data-figure-1/FIGURE-1B/c-cpxar.tif]

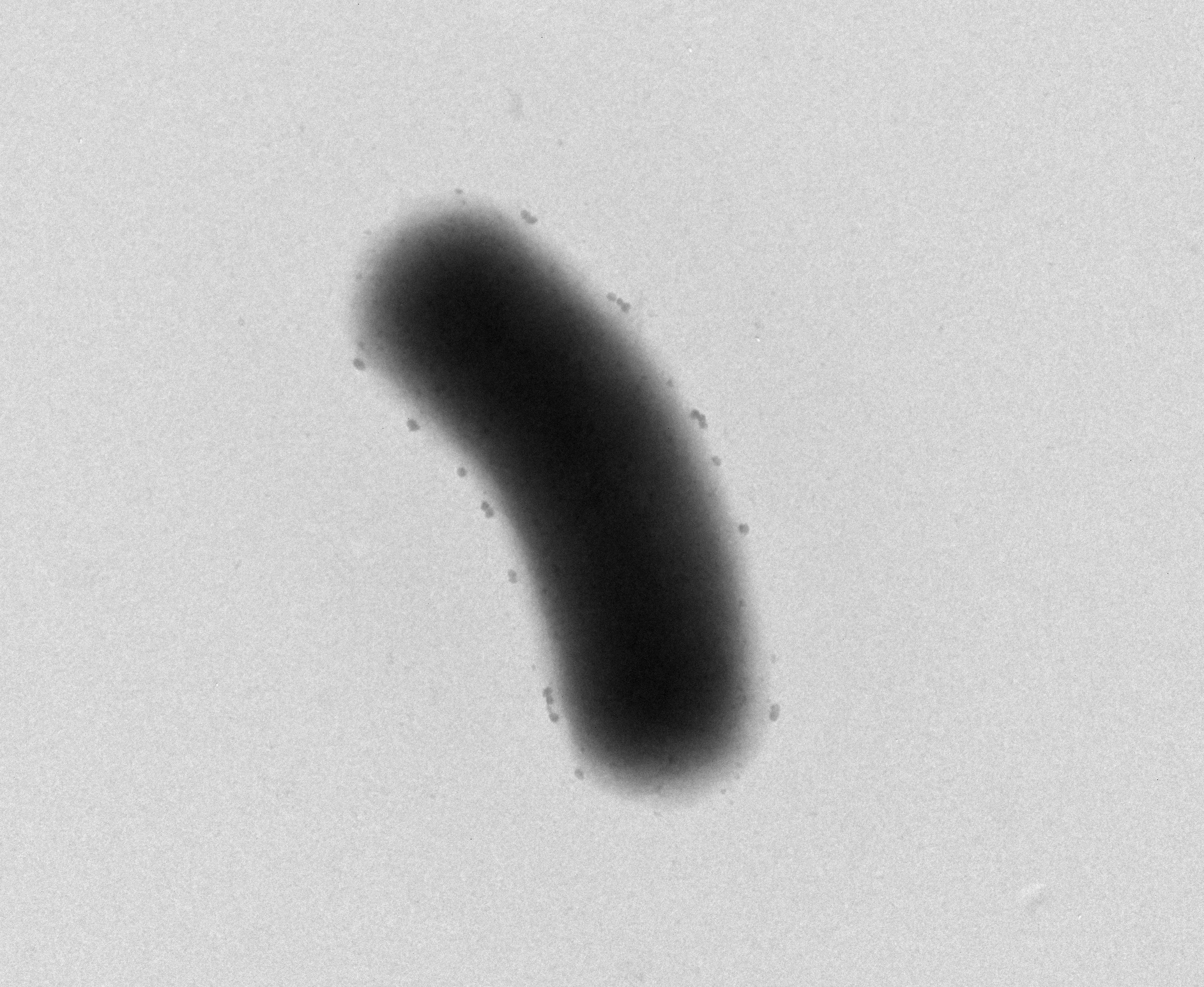

Supplement: Supplementary file 1 [file Data_Sheet_1.ZIP › original data/original data-figure-1/FIGURE-1B/cpxAR.tif]

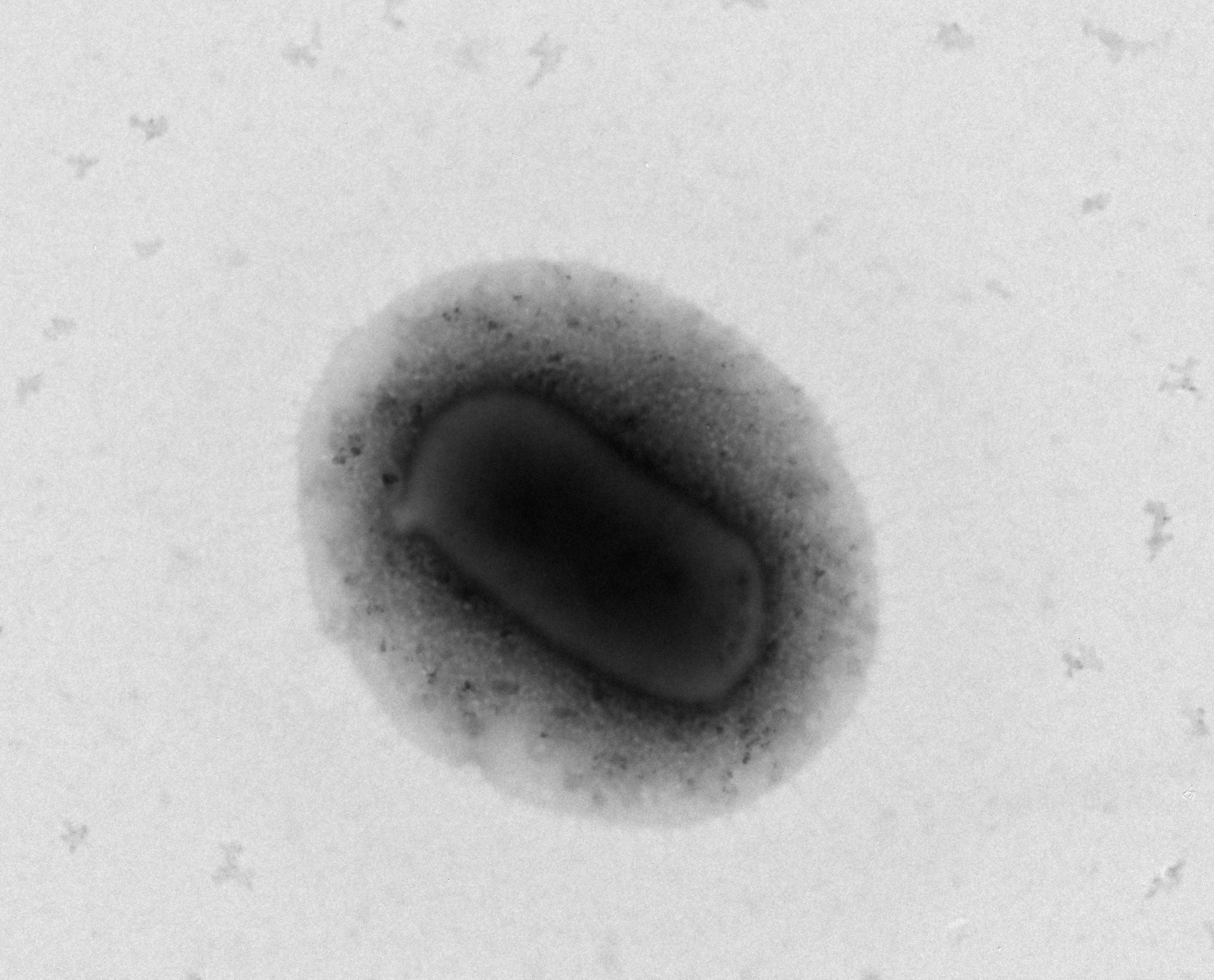

Supplement: Supplementary file 1 [file Data_Sheet_1.ZIP › original data/original data-figure-1/FIGURE-1B/WT.tif]

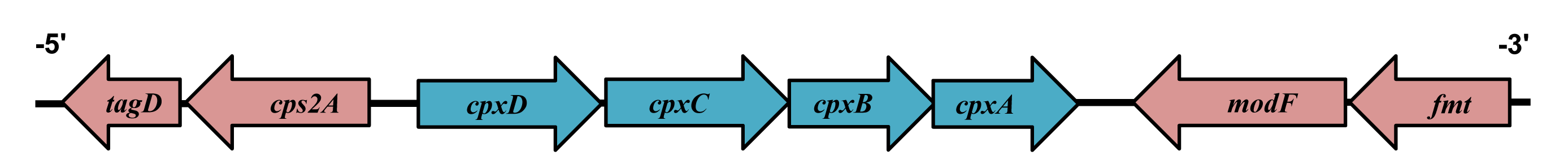

Supplement: Supplementary file 1 [file Data_Sheet_1.ZIP › original data/original data-figure-2/figure-2A/cpxDCBA operon.tif]

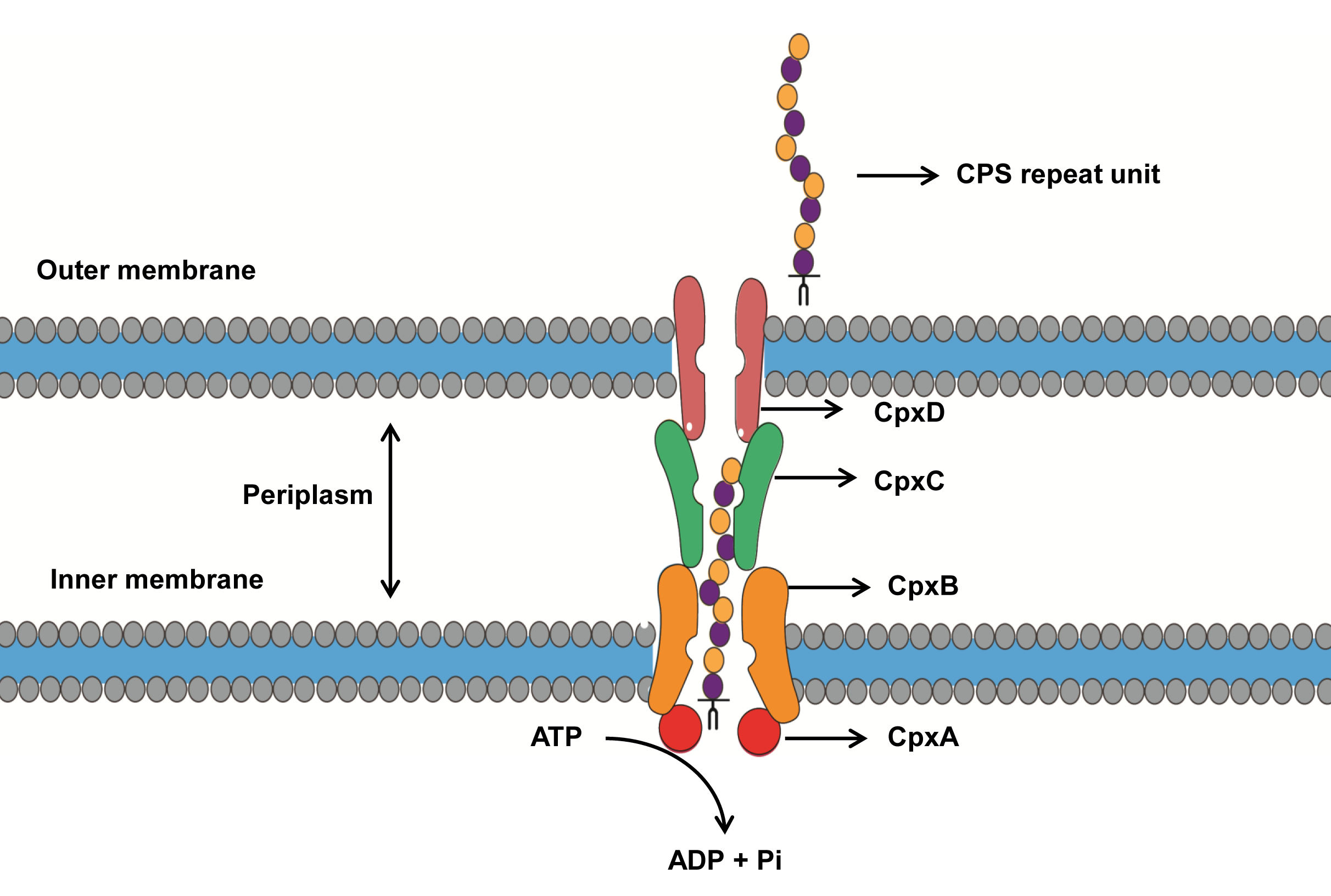

Supplement: Supplementary file 1 [file Data_Sheet_1.ZIP › original data/original data-figure-2/figure-2B/CPS export system.tif]

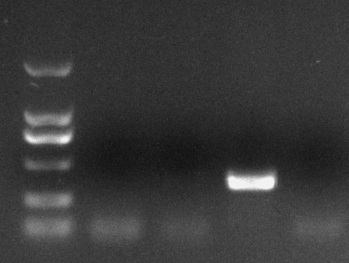

Supplement: Supplementary file 1 [file Data_Sheet_1.ZIP › original data/original data-figure-2/figure-2C/cps2A-cpxD.tif]

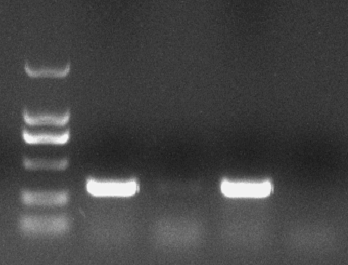

Supplement: Supplementary file 1 [file Data_Sheet_1.ZIP › original data/original data-figure-2/figure-2C/cpxBA.tif]

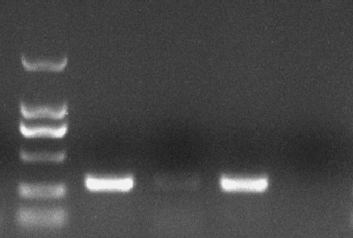

Supplement: Supplementary file 1 [file Data_Sheet_1.ZIP › original data/original data-figure-2/figure-2C/cpxCB.tif]

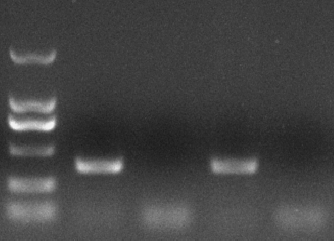

Supplement: Supplementary file 1 [file Data_Sheet_1.ZIP › original data/original data-figure-2/figure-2C/cpxDC.tif]

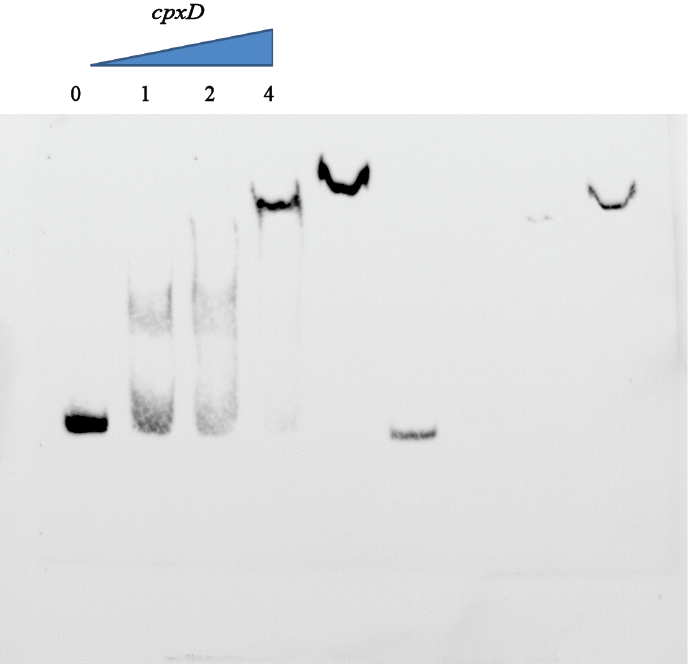

Supplement: Supplementary file 1 [file Data_Sheet_1.ZIP › original data/original data-figure-3/figure-3A/EMSA-cpxD.tif]

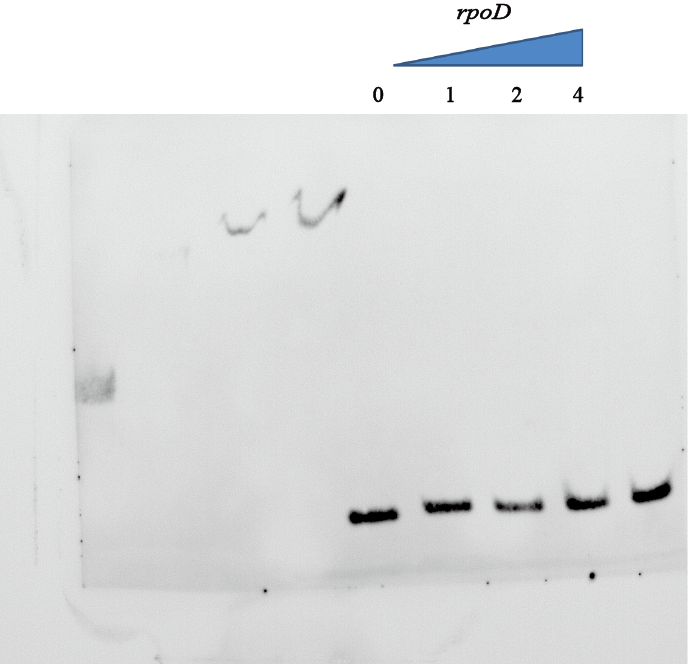

Supplement: Supplementary file 1 [file Data_Sheet_1.ZIP › original data/original data-figure-3/figure-3A/EMSA-rpoD.tif]

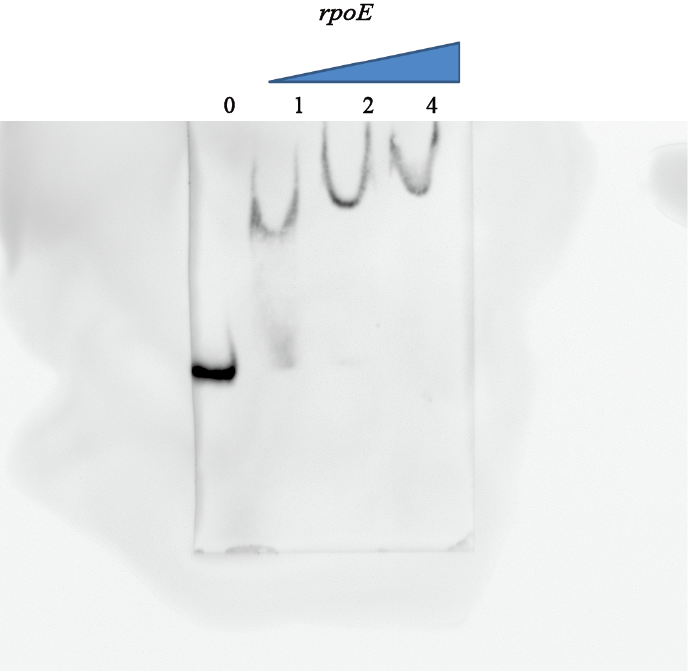

Supplement: Supplementary file 1 [file Data_Sheet_1.ZIP › original data/original data-figure-3/figure-3A/EMSA-rpoE.tif]

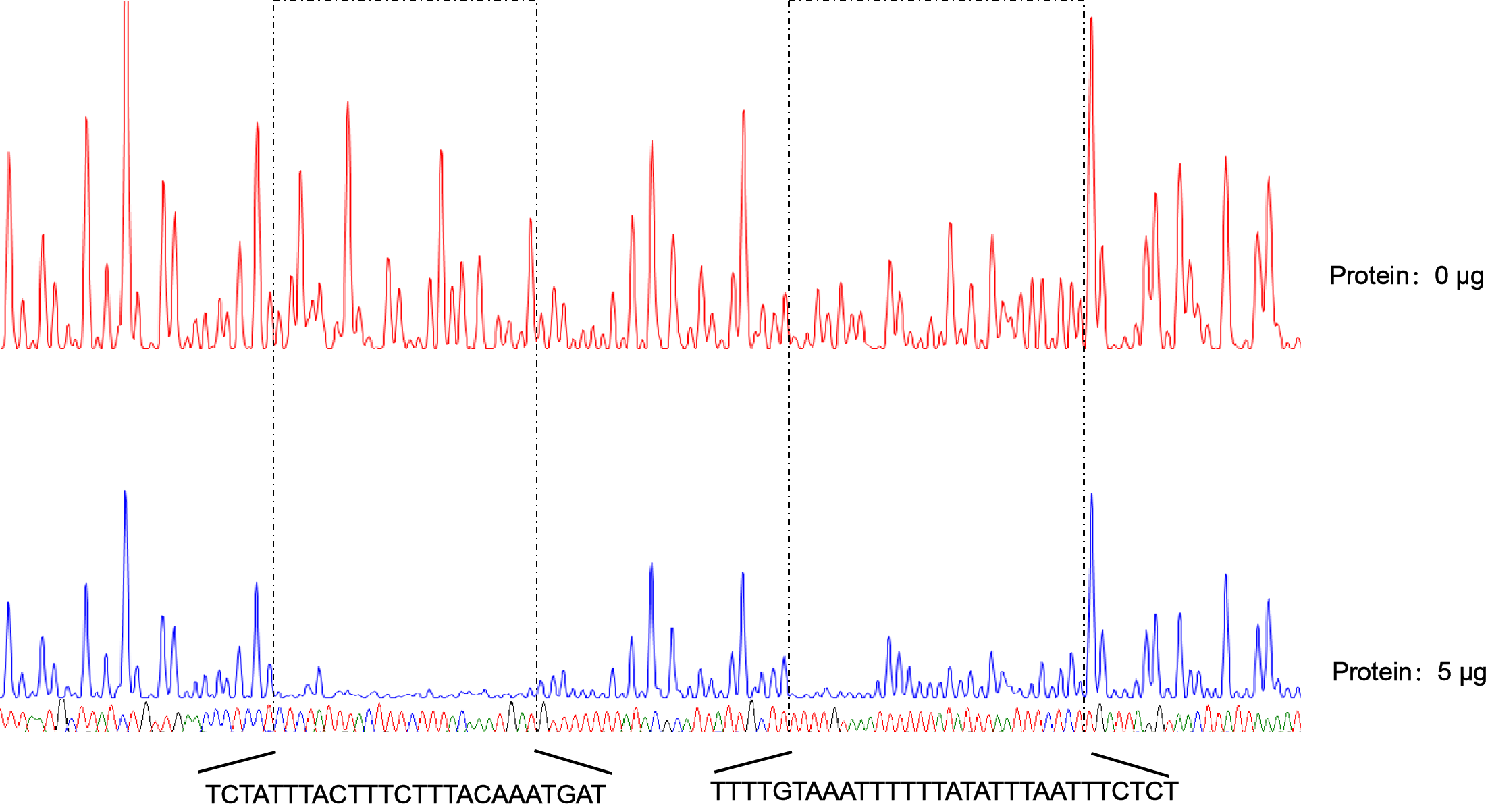

Supplement: Supplementary file 1 [file Data_Sheet_1.ZIP › original data/original data-figure-3/figure-3B/DNaseI footprinting.tif]

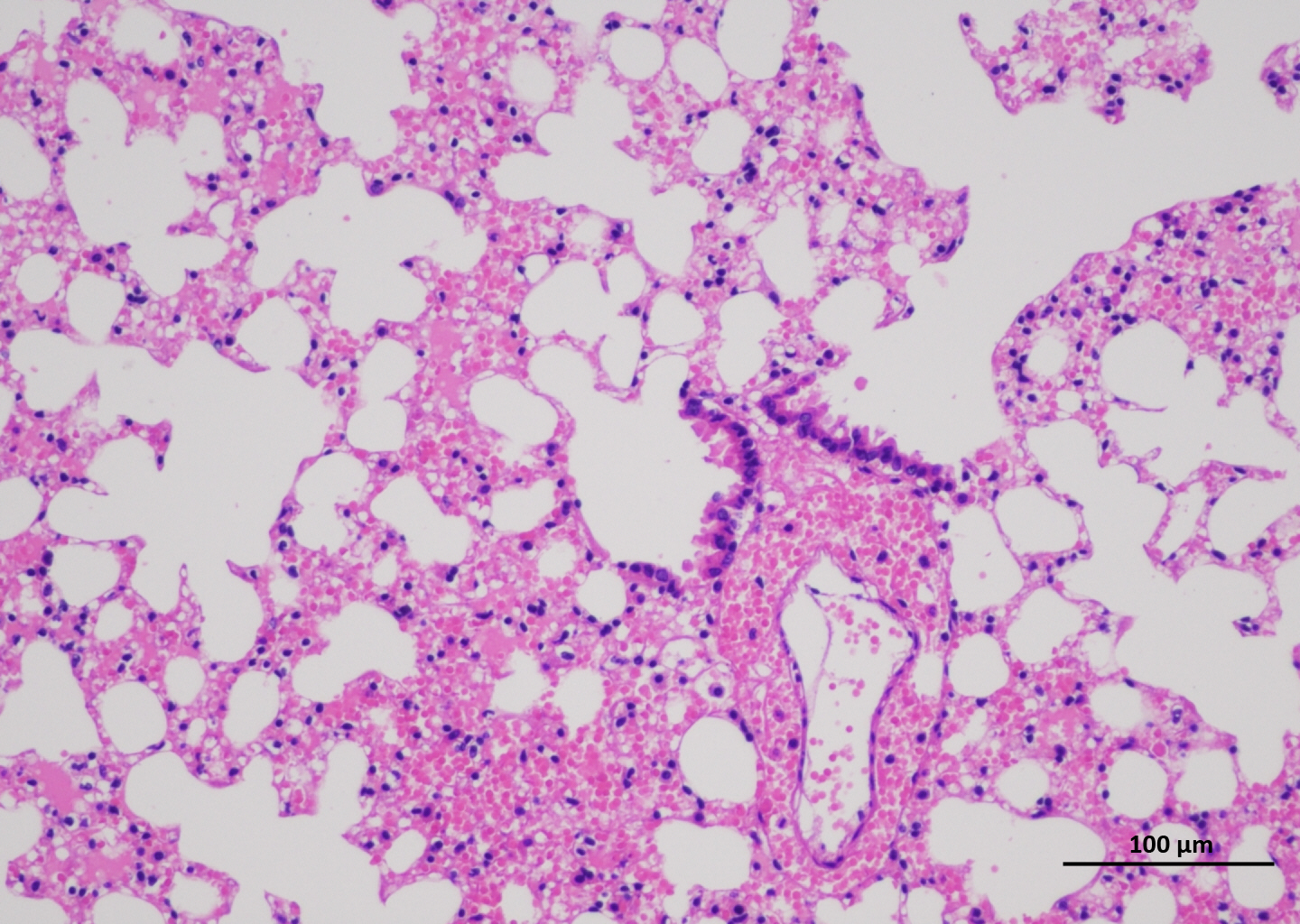

Supplement: Supplementary file 1 [file Data_Sheet_1.ZIP › original data/original data-figure-4/figure-4C/C-cpxAR.jpg]

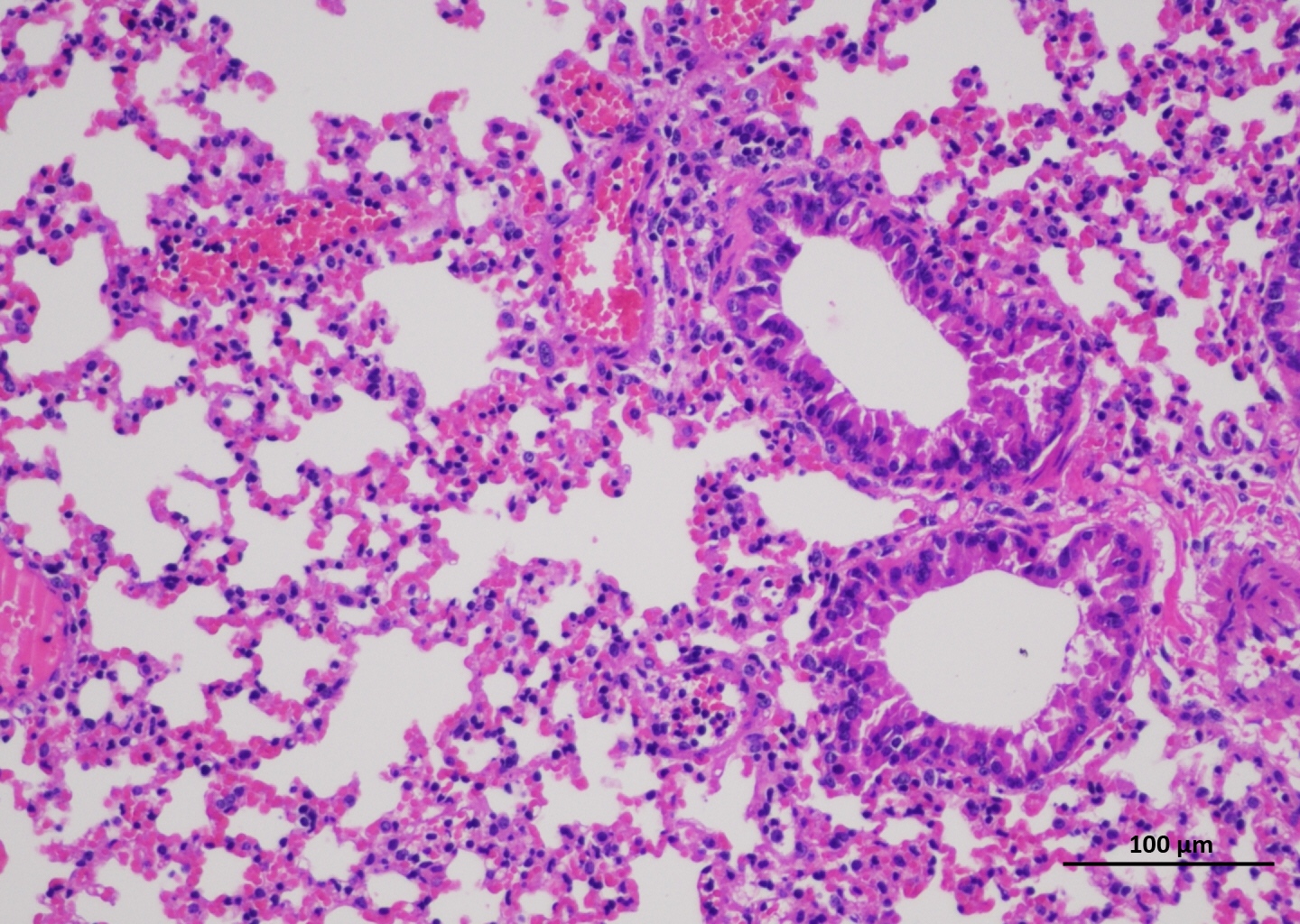

Supplement: Supplementary file 1 [file Data_Sheet_1.ZIP › original data/original data-figure-4/figure-4C/C-cpxD.jpg]

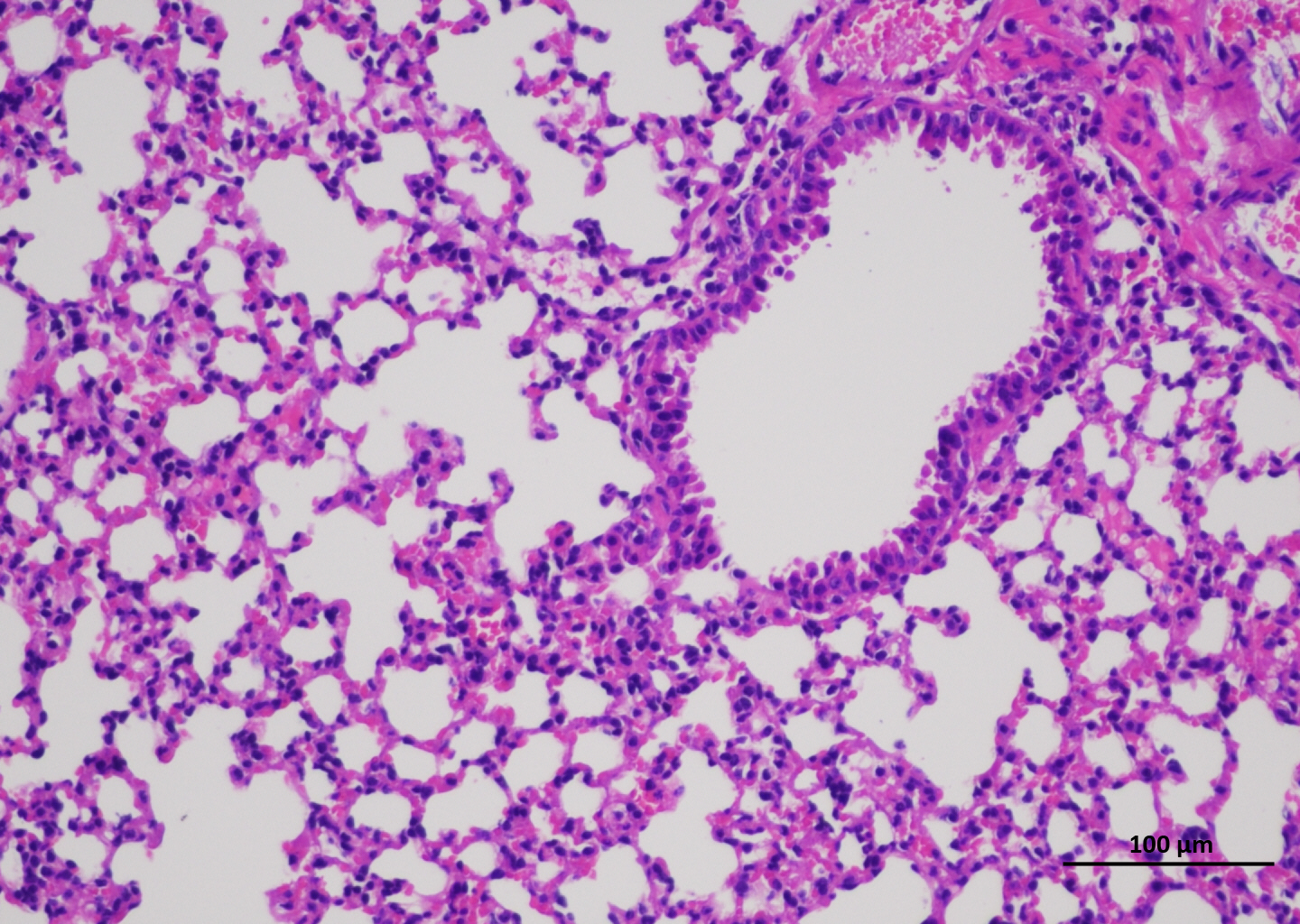

Supplement: Supplementary file 1 [file Data_Sheet_1.ZIP › original data/original data-figure-4/figure-4C/cpxAR.jpg]

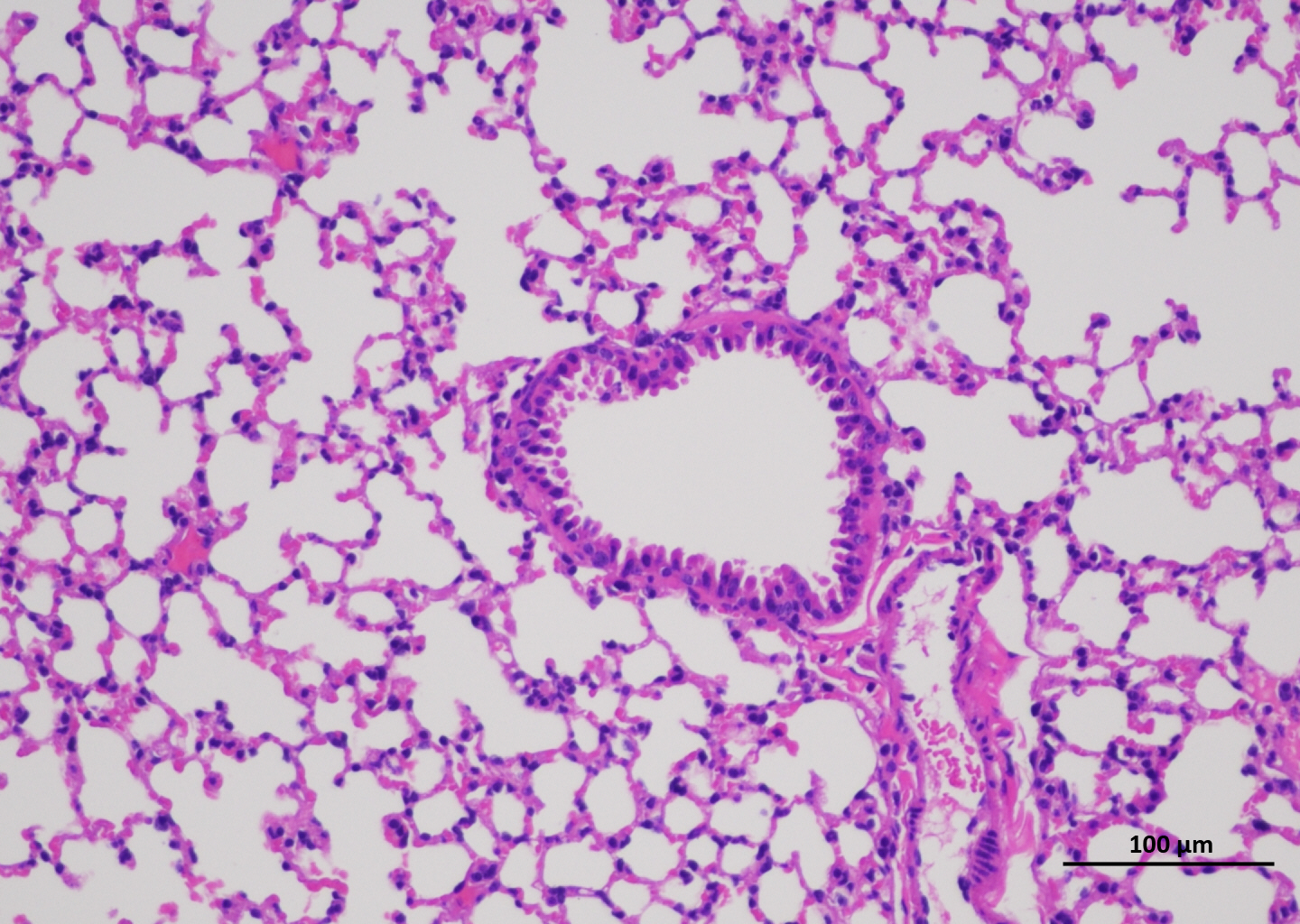

Supplement: Supplementary file 1 [file Data_Sheet_1.ZIP › original data/original data-figure-4/figure-4C/cpxD.jpg]

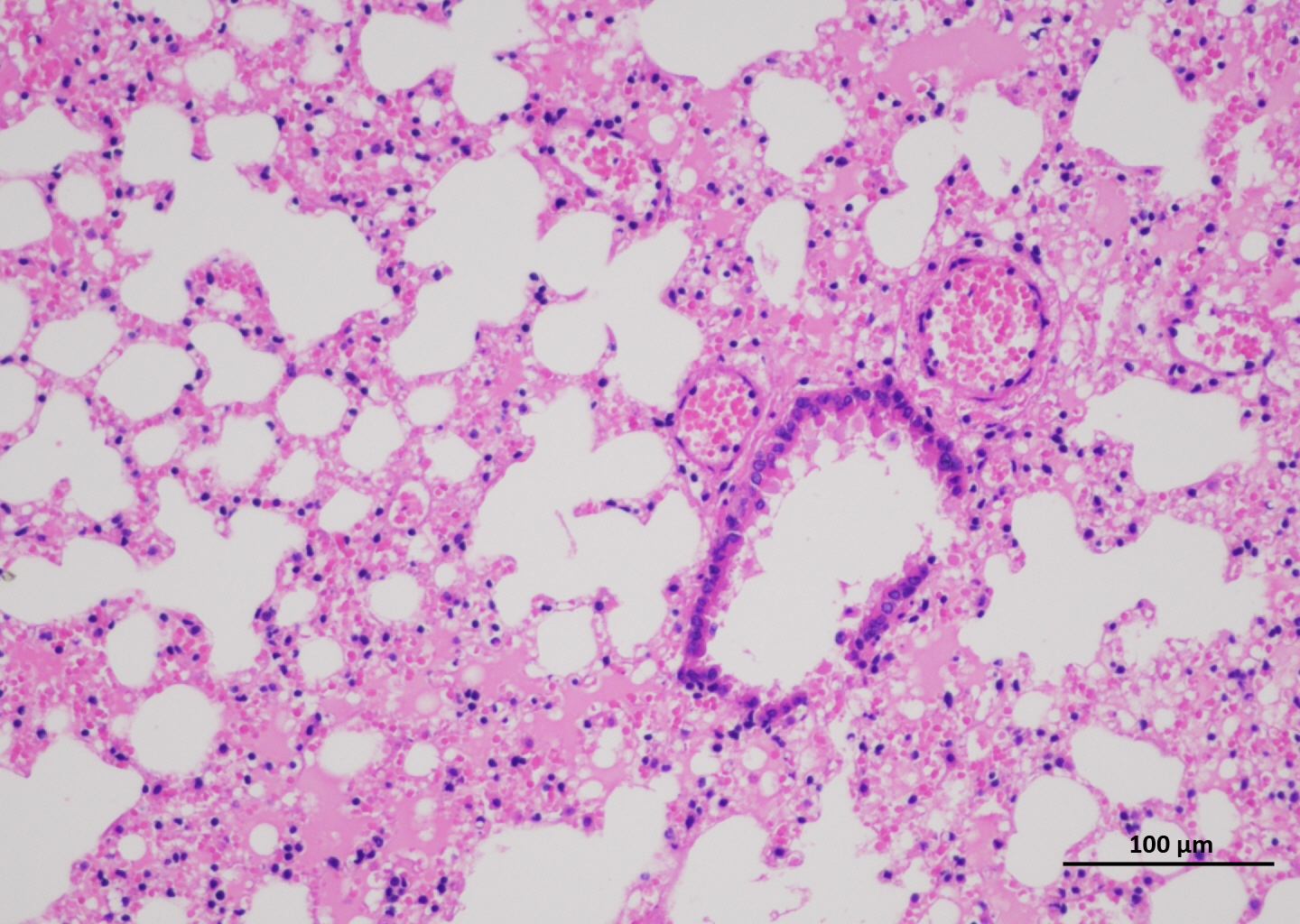

Supplement: Supplementary file 1 [file Data_Sheet_1.ZIP › original data/original data-figure-4/figure-4C/WT.jpg]

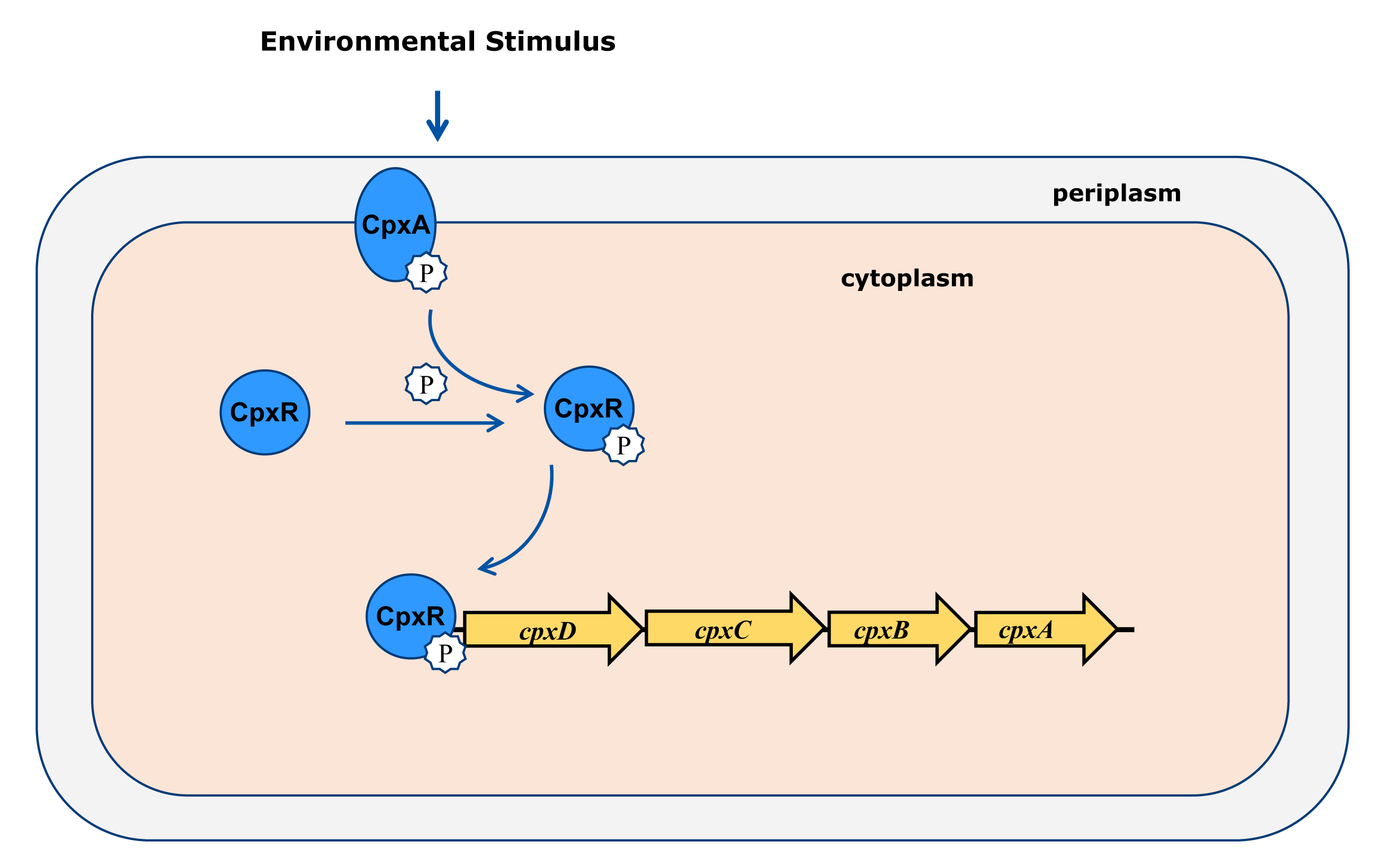

Supplement: Supplementary file 1 [file Data_Sheet_1.ZIP › original data/original data-figure-5/─ú╩╜═╝-1.tif]
